# Assessment of Image Quality and Lesion Detectability With Digital PET/CT System

**DOI:** 10.3389/fmed.2021.629096

**Published:** 2021-02-22

**Authors:** Olivier Delcroix, David Bourhis, Nathalie Keromnes, Philippe Robin, Pierre-Yves Le Roux, Ronan Abgral, Pierre-Yves Salaun, Solène Querellou

**Affiliations:** ^1^Nuclear Medicine Department, Brest University Hospital, Brest, France; ^2^EA 3878 GETBO IFR, Brest, France; ^3^University of Bretagne Occidental, Brest, France

**Keywords:** analog detectors, PET/CT, clinical evaluation, image quality, SiPM

## Abstract

**Purpose:** The aim of this study was to assess image quality and lesion detectability acquired with a digital Positron Emission Tomography/Computed Tomography (PET/CT) Siemens Biograph Vision 600 system.

**Material and Methods:** Consecutive patients who underwent a FDG PET/CT during the first week of use of a digital PET/CT (Siemens Biograph Vision 600) at the nuclear medicine department of the university hospital of Brest were analyzed. PET were realized using list mode acquisition. For all patients, 4 datasets were reconstructed. We determined, according to phantom measurements, an equivalent time acquisition/reconstruction parameters pair of the digital PET/CT corresponding to an analog PET/CT image quality (“analog-like”) as reference dataset. We compared the reference dataset with 3 others digital PET/CT reconstruction parameters, allowing a decrease of emission duration: 60, 90, and 120 s per bed position. Three nuclear medicine physicians evaluated independently, for each dataset, overall image quality [Maximal Intensity Projection (MIP), noise, sharpness] using a 4-point scale. Physicians assessed also lesion detection capability by reporting new visible lesions on each digital datasets with their confidence level in comparison with analog-like dataset.

**Results:** Ninety-eight patients were analyzed. Image quality of MIP (IQ_MIP_), sharpness (IQ_SHARPNESS_), and noise (IQ_NOISE_) of all digital datasets (60, 90, and 120 s) were better than those evaluated with analog-like reconstruction. Moreover, digital PET/CT system improved IQ_MIP_, IQ_NOISE_, and IQ_SHARPNESS_ whatever the BMI. Lesion detection capability and confidence level were higher for 60, 90, 120 s per bed position, respectively, than for analog-like images.

**Conclusion:** Our study demonstrated an improvement of image quality and lesion detectability with a digital PET/CT system.

## Introduction

Positron emission tomography/computed tomography (PET/CT) is a widely used multimodality imaging method that provides metabolic information for oncological and non-oncological disease management (1). Steady improvements in detector design and architecture, as well as implementation of time-of-flight (TOF) and point spread function (PSF) correction technology, have led to significant improvements in sensitivity and image quality (2–5). New type of PET detectors, silicon photomultiplier (SiPM)-based detectors, has been recently developed (6–8) as a key innovation replacing conventional photomultipliers (PMT). Integration of SiPM in PET/CT scanners enabled the development of digital PET/CT scanners, replacing conventional analog PET/CT scanners. The technology, based on semi-conductor detector called SPAD (single photon avalanche diode), has better detection characteristics than PMT. Firstly, if individual SPAD are as sensitive as PMT, SPAD can cover the whole surface of scintillation crystal and the global sensitivity is higher. Secondly, SiPM are faster than PMT, resulting to a time resolution of 215 ps for Siemens Vision PET/CT system (9). The improvement in time resolution lead to more efficient TOF reconstructions with a very fast convergence and noiseless images (10) with high effective sensitivity in regards to PMT systems. Moreover, the sensitivity improvement gave the opportunity to design smaller crystal pixels leading to a better spatial resolution. Based on phantom studies that were recently published using digital PET systems (6, 11), it has been demonstrated that digital PET outperforms analog PET in terms of spatial resolution and sensitivity. However, to our knowledge, data on the comparison of digital PET and analog PET in terms of image quality and diagnostic confidence in patients undergoing PET/CT is scarce (12–14).

The aim of our study was to assess the overall image quality and lesion detection capability improvement in patients undergoing digital PET/CT scanning on a Siemens Biograph Vision 600.

## Materials and Methods

### Patients

All consecutive patients referred for an ^18^F-Fluoro-Deoxy-Glucose (FDG) PET/CT for oncological or non-oncological purpose during the first week of use of the Biograph Vision 600 (Siemens Healthineers, Knoxville, TN, United States) at the nuclear medicine department of Brest university hospital, from Oct 10, 2018 were included. All procedures performed in this study were in accordance with the ethical standards of the institutional research committee on human experimentation and with the Helsinki Declaration of 1975, as revised in 2008. The ethics committee of Brest University Hospital reviewed and approved the protocol of our retrospective study (approval number B2020CE.22). All patients provided written informed consent.

### PET/CT Characteristics

Two PET/CT systems were used: the analog Biograph mCT Flow equipped with extended axial Field Of View (FOV) TrueV and the digital Biograph Vision 600 (Siemens Healthineers, Knoxville, TN, United States).

CT acquisitions and reconstruction parameters were similar for both systems: helical CT acquisition, with iodine contrast when it was possible, was firstly performed for attenuation correction and anatomic localization. CT parameters were 110 kVp (automatic kV modulation, carekV®); 80 refmAs with automatic dose modulation (care4D®); 0.5 second rotation time; 19.2 mm total collimation width; pitch 1, 512 matrix size, 0.98 × 0.98 mm pixels; 2 mm slices thickness.

PET data was acquired in 3D list mode during 120 s per bed position. Axial FOV were 218 and 263 mm and overlap fraction were 43 and 49%, respectively, for Biograph mCT and Biograph Vision.

Images acquired on the Biograph mCT TrueV were reconstructed in daily practice. The reconstruction matrix and voxels size were, respectively, 200 × 200 and 4 × 4 × 2 mm and the reconstruction algorithm was OSEM3D with application of TOF and resolution modeling, 2 iterations, 21 subsets, and 2 mm Gaussian post filtering.

Images acquired on the Biograph Vision 600 were reconstructed in daily practice. The reconstruction matrix and voxels size were, respectively, 440 × 440 and 1.65 × 1.65 × 1.65 mm and the reconstruction algorithm was OSEM3D with application of TOF and resolution modeling, 4 iterations, 5 subsets, and 2 mm Gaussian post filtering.

### Phantom PET/CT Preclinical Study

In order to avoid undergoing 2 consecutive PET/CT on 2 different systems (analog and digital systems) for the evaluation of image quality and lesion detectability, we performed a preclinical study using phantom filled with FDG. Phantom was scanned on both analog and digital systems to estimate the acquisition and reconstruction parameters providing an image quality comparable to an analog system (“Analog-like”) acquired on digital PET/CT and then used these data for the clinical study with a single acquisition on a digital PET/CT system alone.

So, we determined, according to phantom measurements, an equivalent time acquisition/reconstruction parameters pair of the digital PET/CT Biograph Vision 600 corresponding to an analog PET/CT image quality (“analog-like”) of the Biograph mCT TrueV as reference.

A NEMA IEC phantom, filled with 5 MBq/L in the background compartment and 20 MBq/L in the spheres (diameters were, respectively, 13, 17, 21, 28, and 37 mm), was both scanned on a Biograph mCT and a Biograph Vision during 120 s in list-mode. According to the literature, NEMA phantom measurements on Biograph mCT and on Biograph Vision systems demonstrated a sensitivity ratio of 1.6 (11, 15). TOF effective sensitivity gain ratio was estimated by Δt_mCT_/Δt_Vision_ = 2.5 (16). Thus, the overall sensitivity ratio between Biograph mCT and Biograph Vision is ~1.6 × 2.5 = 4, allowing to determine 30 s emission time per bed position for the Biograph Vision in comparison with 120 s for the Biograph mCT. On this basis, multiple reconstruction parameters were tested on the Biograph Vision and compared to the results obtained with the Biograph mCT. On reconstructed images, Contrast Recovery (CR) was calculated as the ratio of the image contrast (ratio of mean intensity in spheres and mean intensity in background) and the radioactivity contrast (4:1). Analog-like reconstruction parameters on the Biograph Vision were selected in order to simulate an equivalent CR on the Biograph mCT.

We also determined the reconstruction parameters that would allow to perform 60 and 90 s per bed position compared to the daily practice reference on Biograph Vision (120 s per bed position, matrix of 440 × 440 with voxels size of 1.65 × 1.65 × 1.65 mm, OSEM3D with application of TOF and resolution modeling, 4 iterations, 5 subsets, and 2 mm Gaussian post filtering).

Multiple reconstructions were performed and signal to noise ratio (SNR) was calculated as the ratio of the standard deviation and the mean intensity value in six regions of interest in the background area. Sixty and ninety seconds protocols reconstruction parameters were determined in such a way that SNR was at least equivalent to the daily practice reference reconstruction and images as sharp as possible.

### Patients PET/CT Clinical Study

Prior to radiotracer injection, all patients fasted for at least 6 h and then were injected with standard activity of ^18^F-FDG (3 MBq/kg). Approximately 60 min after FDG injection (17), patients underwent a list-mode PET/CT imaging protocol only on the Biograph Vision. PET data was reconstructed according to “analog-like,” 60, 90, and 120 s protocols.

### Image Analysis

Images were reviewed using a dedicated workstation (Syngovia, VB30, Siemens Healthineers) with 4 PET datasets opened and linked automatically for their simultaneous assessment. Image quality was assessed independently by three experienced nuclear medicine physicians (OD, NK, and SQL with two, 8 and 15 years of experience in PET reading, respectively). Interpreters were blinded of all clinical data but were aware of the subset of reconstruction data (“analog-like” dataset, 60, 90, and 120 s per bed position digital datasets). Analysis of image quality was performed in two steps.

The first step was an overall qualitative assessment using a four-point scale (ranging from a score of 1 [excellent quality maximum intensity projection (MIP)] to 4 [poor quality MIP]) (18). The 4 datasets were subsequently compared side by side for noise and sharpness with the same scale (18). Previous criteria were also classified according to body mass index (BMI) for each PET datasets on the basis of the patient's BMI (BMI < 25 kg/m^2^; BMI > 25 kg/m^2^).

The second step consisted for each physician to identify any new FDG uptake in each digital dataset, in comparison with the “analog-like” dataset. For each uptake identified, physicians gave their confidence level according to a four-point scale (1[high confidence] to 4[low confidence]).

### Statistical Analysis

Each parameter (overall quality of MIP, noise, and sharpness) was calculated for each reader and for all readers [mean +/– standard deviation (SD)] according to reconstruction datasets and on the basis of patients' BMI.

Data is presented as mean values +/– SD. The Wilcoxon test was used to compare data according to each reconstruction and BMI subgroups (BMI < 25 kg/m^2^; BMI > 25 kg/m^2^). A *p* < 0.05 was considered statistically significant. Statistical analysis was performed using XLSTAT-Life software (Addinsoft, Paris, France).

## Results

### Phantom Study

**Figure 1:**
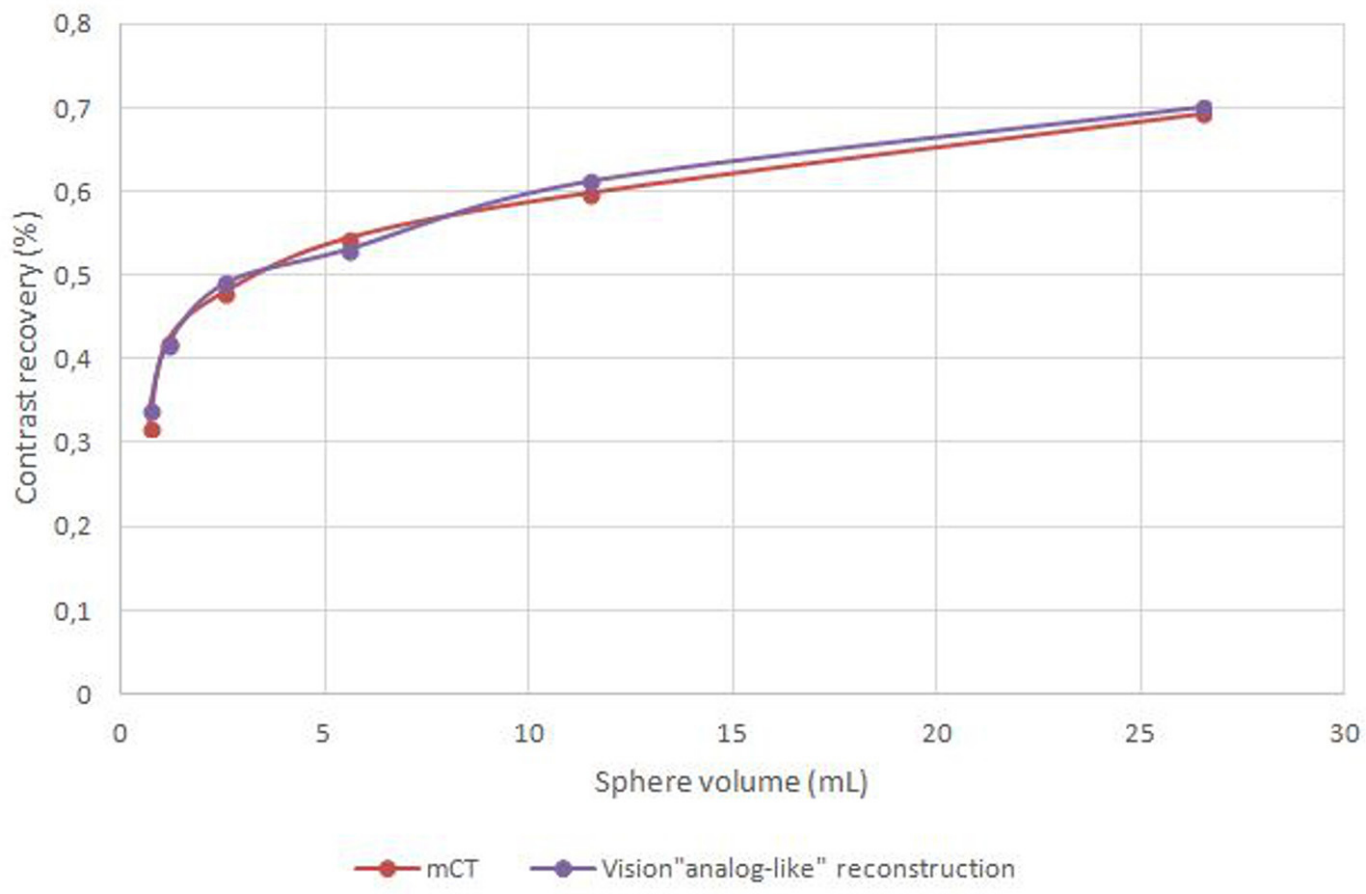
Contrast recovery measured on original Biograph mCT images and on Biograph Vision “Analog like” images.

The reconstruction parameters which produced the best Analog like reconstruction were 3.3 × 3.3 × 1.65 mm voxels, 5 iterations, 5 subsets and 4 mm Gaussian post filtering. CR measured on original Biograph mCT images and Analog like Biograph Vision reconstruction are shown in Figure 1.

Reconstruction parameters and the corresponding SNR are detailed in Table 1.

**Table 1:** Vision reconstruction parameters and the corresponding SNR.

**Frame duration**	**Parameters**	**SNR**
Analog like	3.3 × 3.3 × 1.65 mm voxels, 5 iterations, 5 subsets, 4 mm Gaussian filter	10.8
60 s	1.65 × 1.65 × 1.65 mm voxels, 3 iterations, 5 subsets, 3 mm Gaussian filter	9.7
90 s	1.65 × 1.65 × 1.65 mm voxels, 3 iterations, 5 subsets, 2 mm Gaussian filter	9.6
120 s	1.65 × 1.65 × 1.65 mm voxels, 4 iterations, 5 subsets, 2 mm Gaussian filter	9

### Patients Study

Ninety-nine patients were included. One patient was excluded from the analysis because of missing data.

Characteristics of the remaining 98 patients are summarized in Table 2.

**Table 2:** Patients characteristics.

**Characteristics**	**No. of patients (%) (*n =* 98)**
Age (years)	
Mean ± SD	64 ± 13
Median (range)	65 (19-88)
Sex—no. (%)	
Male	58 (59%)
Female	40 (41%)
Weight (kg)	
Mean ± SD	73 ± 19
Median (range)	70 (45–140)
BMI	
Mean ± SD	25.5 ± 6.1
Median (range)	24.3 (15.6–49.6)
FDG activity injected (MBq)	
Mean ± SD	222 ± 58
Median (range)	212 (134–425)
Delay between injection and acquisition (min)	
Mean ± SD	61.2 ± 5.7
Median (range)	60 (55–94)
Glucose blood level (mmol/L)	
Mean ± SD	5.9 ± 1
Median (range)	5.7 (4.3–10.4)
Iodine contrast CT—no. (%)	
Yes	63 (64%)
No	35 (36%)

### Whole-Body Image Quality Analysis

Data of overall qualitative assessment (MIP, noise, and sharpness) is detailed below in Table 3.

**Table 3:** Data of overall qualitative assessment (MIP, noise, and sharpness) according to readers.

	**Reader 1**		**Reader 2**		**Reader 3**		**Total**		
**MIP**	**Mean-value**	***SD***	**Mean-value**	***SD***	**Mean-value**	***SD***	**Mean-value**	***SD***	***p***
Analog like	2.14	0.41	2.44	0.54	2.34	0.48	2.31	0.49	
60 s	1.16	0.40	1.46	0.54	1.26	0.44	1.29	0.48	<0.0001
90 s	1.11	0.35	1.10	0.30	1.14	0.35	1.12	0.33	<0.0001
120 s	1.09	0.32	1.08	0.28	1.13	0.34	1.10	0.31	<0.0001
**Noise**	**Mean-value**	***SD***	**Mean-value**	***SD***	**Mean-value**	***SD***	**Mean-value**	***SD***	***p***
Analog like	2.20	0.45	2.51	0.60	2.33	0.51	2.35	0.54	
60 s	1.19	0.42	1.39	0.57	1.19	0.40	1.26	0.48	<0.0001
90 s	1.10	0.34	1.12	0.33	1.09	0.29	1.11	0.32	<0.0001
120 s	1.11	0.32	1.18	0.41	1.17	0.38	1.16	0.37	<0.0001
**Sharpness**	**Mean-value**	***SD***	**Mean-value**	***SD***	**Mean-value**	***SD***	**Mean-value**	***SD***	***p***
Analog like	2.86	0.35	2.31	0.51	2.48	0.50	2.55	0.51	
60 s	1.91	0.29	1.88	0.39	1.56	0.50	1.78	0.43	<0.0001
90 s	1.78	0.42	1.13	0.34	1.18	0.39	1.36	0.48	<0.0001
120 s	1.04	0.20	1.05	0.22	1.07	0.26	1.05	0.23	<0.0001

**Figure 2:**
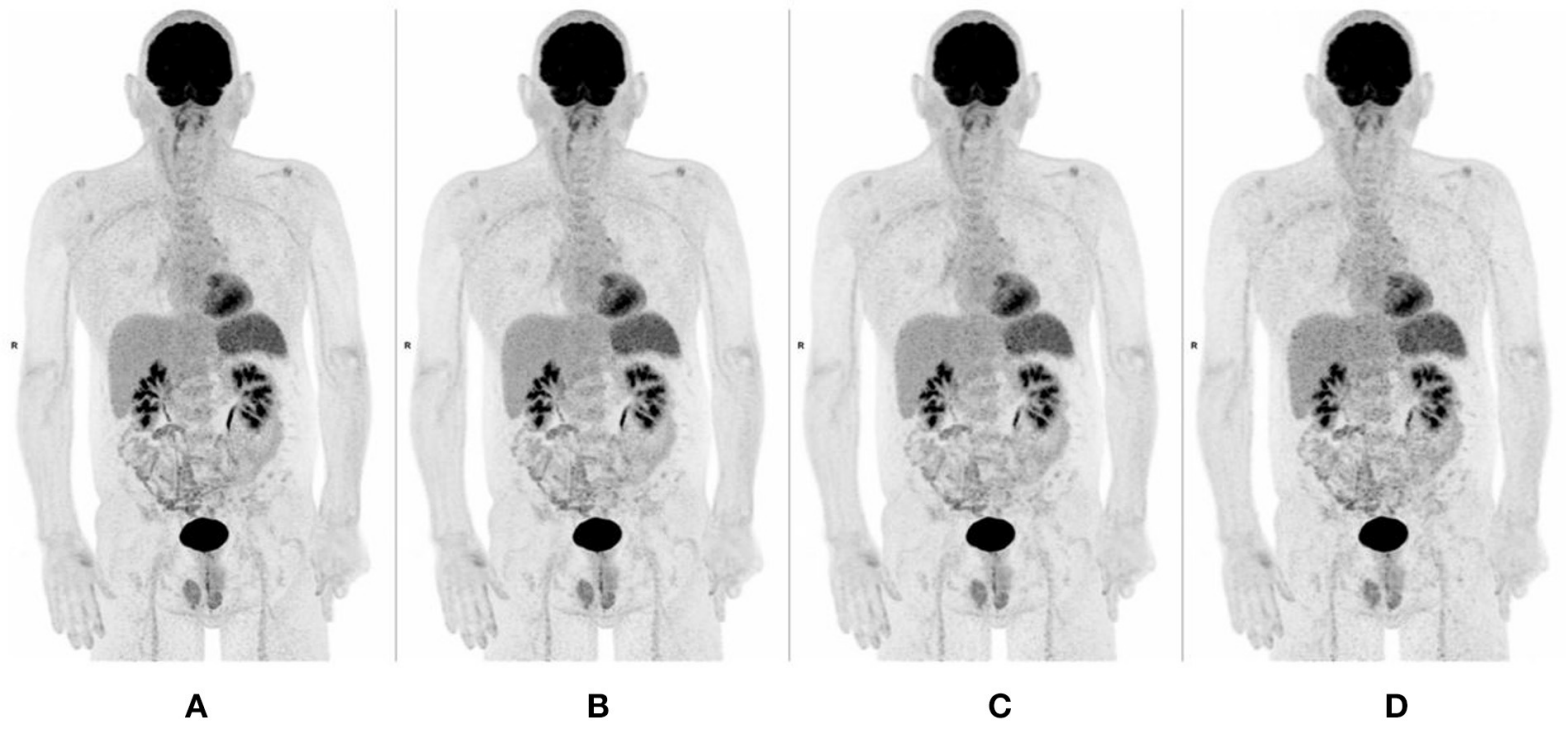
MIP images of a 61 years old man, showing improvement of quality, sharpness, and noise when increasing acquisition time [**(A-D)** from left to right, respectively, 120, 90, 60, and 30 s per bed position].

**Figure 3:**
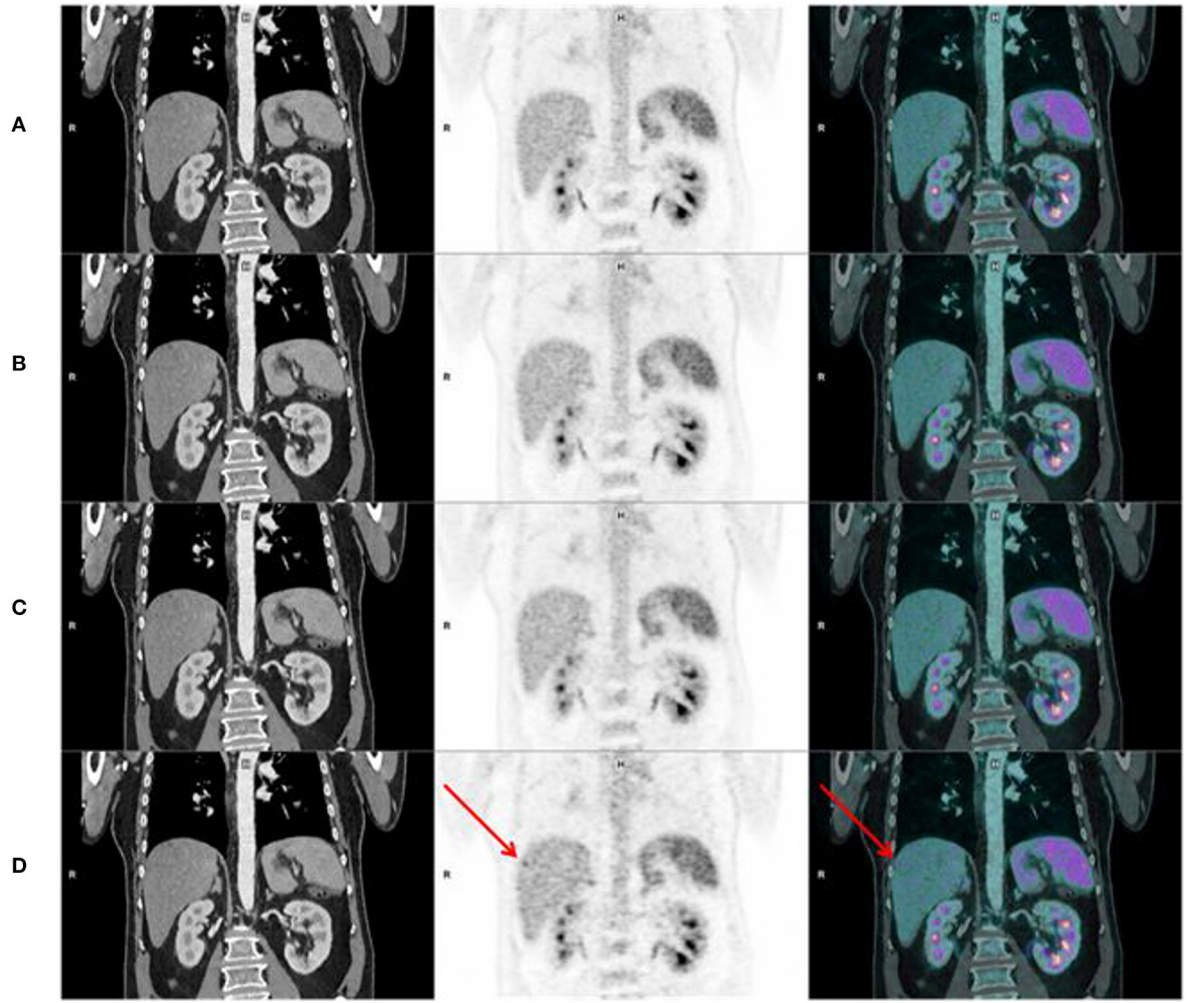
Coronal CT, PET, and fusion images in the same patient, showing better sharpness of organs, pyelo-ureteral uptakes, and less hepatic noise when increasing acquisition time [**(A-D)** from top to bottom, respectively, 120, 90, 60, and 30 s per bed position]. We can see noise on the “Analog-like” acquisition which can mimic focal uptake in the right liver (arrow), and which disappears on the other acquisitions.

**Figure 4:**
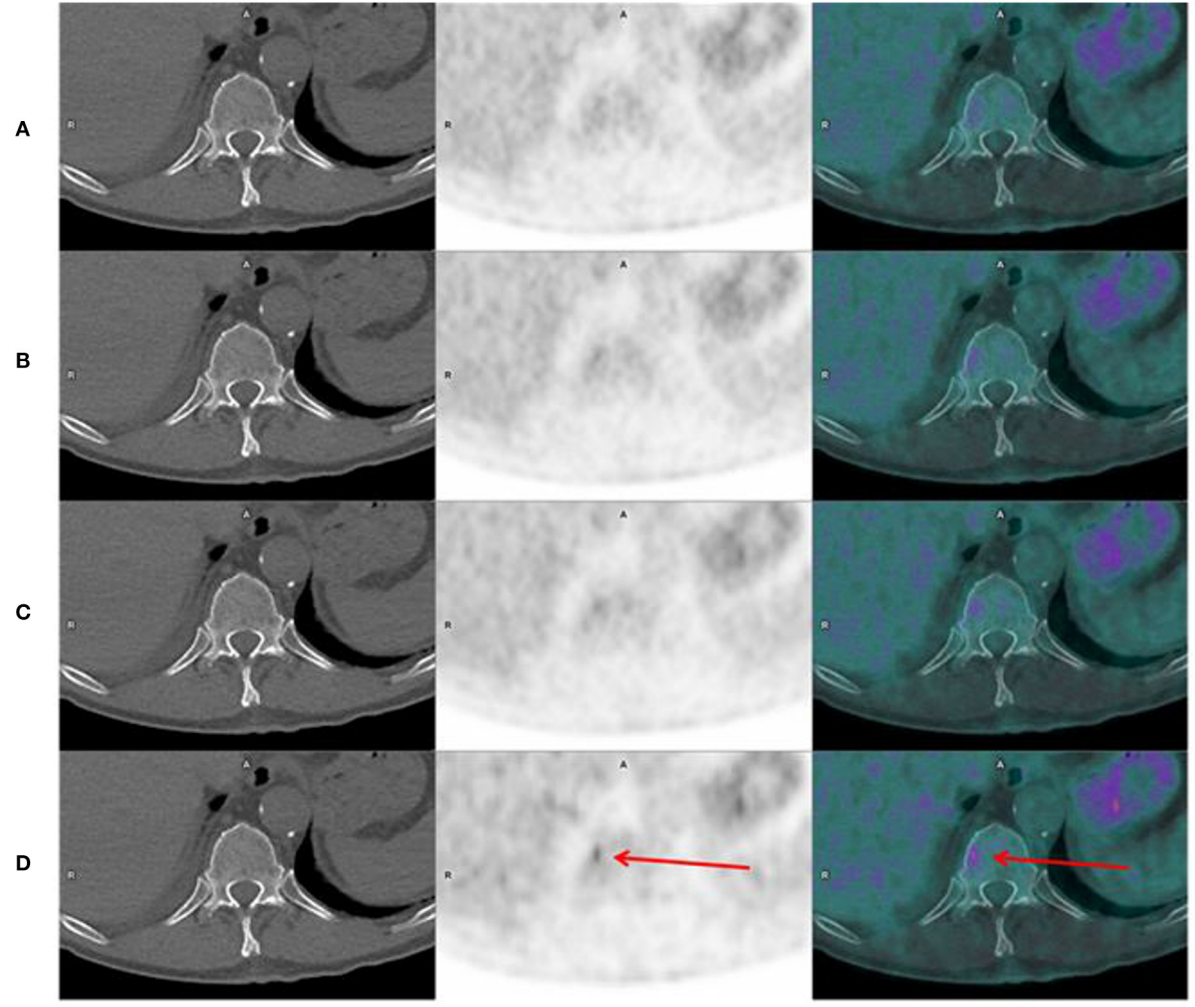
Axial CT, PET, and fusion images in a 65 years old man, showing a bone focal uptake in T11, on the “Analog-like” acquisition, which disappears when increasing acquisition time, and corresponding actually to noise [**(A-D)** from top to bottom, respectively, 120, 90, 60, and 30 s per bed position].

For all readers, image quality of MIP (IQ_MIP_), sharpness (IQ_SHARPNESS_), and noise (IQ_NOISE_) of all digital datasets (60, 90, and 120 s per bed position) were better than those evaluated with “analog-like” reconstruction: MIP was 1.10 ± 0.31 for the 120 s per bed position dataset as compared with 2.31 ± 0.49 for the “analog-like” dataset. Longer acquisition duration per bed position was associated with better IQ_MIP_ and IQ_SHARPNESS_ indices as illustrated in Figures 2–4.

The analysis according to BMI (BMI < 25 kg/m^2^, BMI > 25 kg/m^2^) is described below in Table 4.

**Table 4:** Data of overall qualitative assessment (MIP, noise, and sharpness) according to BMI (< or > 25 kg/m^2^).

	**BMI < 25 (56 patients)**		**BMI >25 (42 patients)**	
**MIP**	**Mean-value**	***SD***	**Mean-value**	***SD***
Analog like	2.19	0.41	2.46	0.54
60 s	1.19	0.41	1.43	0.53
90 s	1.08	0.29	1.17	0.37
120 s	1.04	0.22	1.19	0.39
**Noise**	**Mean-value**	***SD***	**Mean-value**	***SD***
Analog like	2.17	0.41	2.58	0.59
60 s	1.15	0.36	1.40	0.57
90 s	1.04	0.19	1.19	0.42
120 s	1.05	0.21	1.30	0.48
**Sharpness**	**Mean-value**	***SD***	**Mean-value**	***SD***
Analog like	2.48	0.50	2.64	0.51
60 s	1.74	0.44	1.79	0.45
90 s	1.34	0.47	1.39	0.49
120 s	1.03	0.17	1.09	0.28

In BMI subgroups IQ_MIP_, IQ_SHARPNESS_, and IQ_NOISE_ improved with the acquisition duration except for three comparisons. These results are detailed below in Table 5.

**Table 5:** Statistical analysis of parameters (MIP, noise, and sharpness) according to datasets for patients under or over 25 kg/m^2^.

		**MIP**			**Noise**			**Sharpness**		
		**Analog like**	**60**	**90**	**Analog like**	**60**	**90**	**Analog like**	**60**	**90**
BMI < 25 56 patients	Analog like									
	60	<0.0001			<0.0001			<0.0001		
	90	<0.0001	<0.0001		<0.0001	<0.0001		<0.0001	<0.0001	
	120	<0.0001	<0.0001	0.0078	<0.0001	<0.0001	0.4237	<0.0001	<0.0001	<0.0001
BMI > 25 42 patients	Analog like									
	60	<0.0001			<0.0001			<0.0001		
	90	<0.0001	0.0004		<0.0001	<0.0001		<0.0001	<0.0001	
	120	<0.0001	0.0009	0.1489	<0.0001	0.0004	0.0004	<0.0001	<0.0001	<0.0001

IQ_MIP_ was not statistically better for the 120 s reconstruction dataset than for the 90 s reconstruction dataset in patients with BMI > 25 kg/m^2^. IQ_NOISE_ was not statistically better for the 120 s reconstruction dataset than for the 90 s reconstruction dataset in patients with BMI < 25 kg/m^2^ and IQ_NOISE_ was statistically better for the 90 s reconstruction dataset than for the 120 s reconstruction dataset for patients with BMI > 25 kg/m^2^.

According to dichotomy BMI analysis under or over 25 kg/m^2^, IQ_MIP_, IQ_NOISE_ and IQ_SHARPNESS_ were better for patients with a BMI < 25 kg/m^2^ than for patients with a BMI > 25 kg/m^2^, for all reconstruction datasets. These results are detailed below in Table 6.

**Table 6:** Statistical analysis of parameters (MIP, noise, and sharpness) according to BMI for all datasets.

	**BMI <25 vs. BMI>25**								
	**MIP**			**Noise**			**Sharpness**		
	** <25**	**>25**	***p***	** <25**	**>25**	***p***	** <25**	**>25**	***p***
Analog like (30)	2.19	2.46	<0.0001	2.17	2.58	<0.0001	2.48	2.64	0.007
60	1.19	1.43	<0.0001	1.15	1.40	<0.0001	1.74	1.79	0.284
90	1.08	1.17	0.019	1.04	1.20	<0.0001	1.34	1.40	0.311
120	1.04	1.19	<0.0001	1.05	1.30	<0.0001	1.03	1.09	0.032

IQ_SHARPNESS_ was not statistically different between the 60 and the 90 s reconstruction datasets (*p* = 0.284 and *p* = 0.311, respectively).

### Lesion Detectability

**Figure 5:**
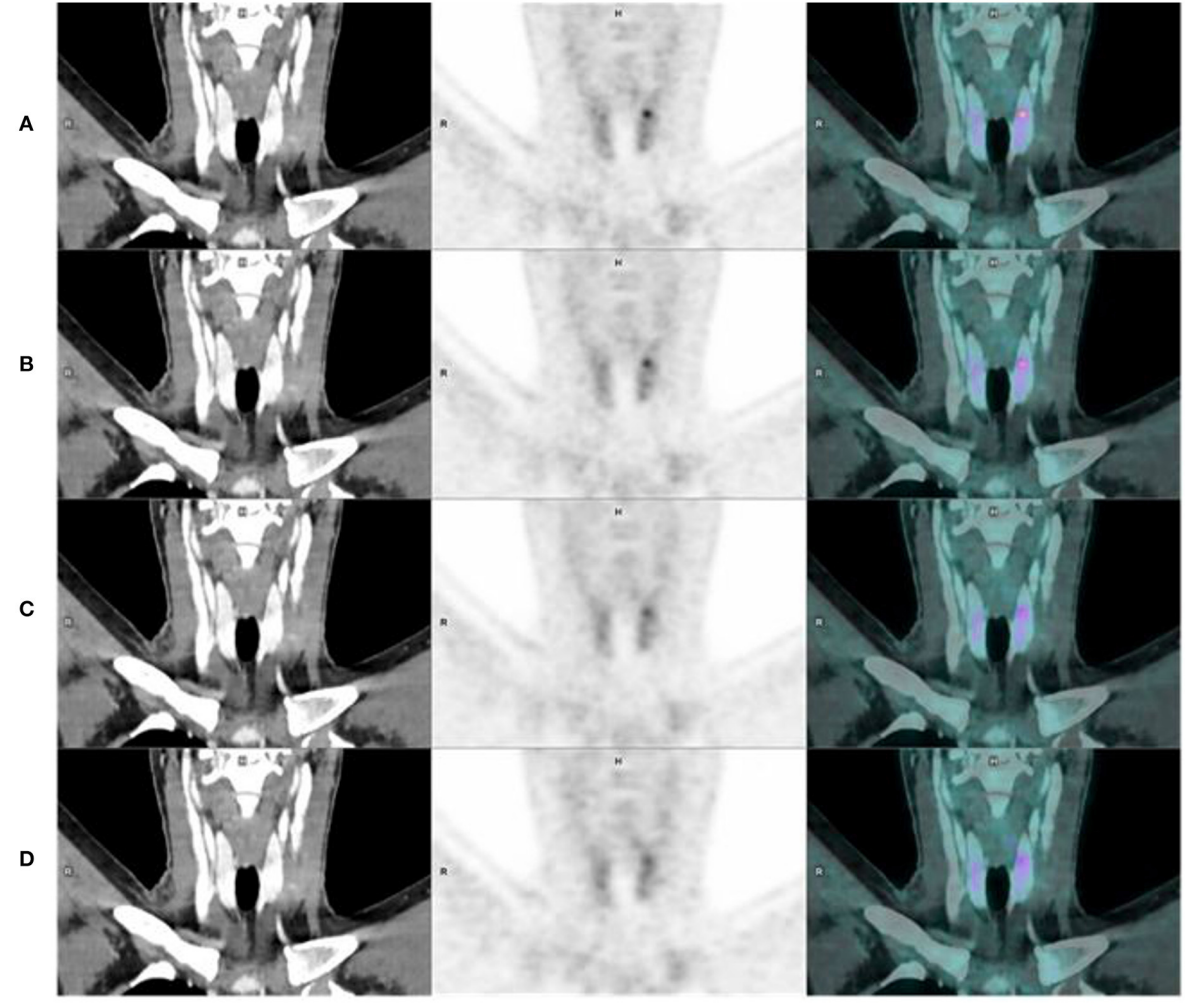
Coronal CT, PET, and fusion images in a 48 years old woman, illustrating detectability improvement when increasing acquisition time, with appearance of a focal uptake in left thyroid lobe, which cannot be seen on the “Analog-like” acquisition [**(A-D)** from top to bottom, respectively, 120, 90, 60, and 30 s per bed position].

The detectability improved progressively with the acquisition per bed position duration as illustrated in Figures 4, 5. These results are presented below in Table 7.

**Table 7:** Analysis of new uptake and confidence level of all readers according to datasets.

**Physicians (SQ, OD, and NK)**		
**Dataset**	**All new uptake (vs. Analog-like)**	**Confidence Level**
60 s	146	2.38
90 s	189	1.85
120 s	201	1.49

Longer the acquisition step bed duration was, higher was the number of detected lesions. The confidence index improved also progressively with the acquisition bed position duration.

## Discussion

Our study showed that digital PET/CT provides improved image quality and outperforms analog PET/CT, especially for MIP, sharpness, and noise with increased detectability at equal or less acquisition duration and injected activity. Up to date, only few studies evaluated in clinical practice, the differences of image quality and lesion detectability between digital and analog PET/CT systems. According to phantom measurements, several studies highlighted a quality improvement of digital PET/CT on a variety of commercial systems: Discovery MI (8), Vereos (6), and Vision (11). Gnesin et al. (19) compared the image quality in 5 PET/CT systems (3 digital PET/CT and 2 analog devices) and reported that a comparable image quality is achievable with a TAP [time (min) ^*^ mass activity (MBq/kg) product] reduction of 40% with digital PET/CT. Moreover, the authors suggested that digital PET systems potentially lead to lesions detectability improvement. We assumed in our study a time reduction factor of 4 between Biograph mCT and Biograph Vision. This was consistent with previous articles on phantom studies: indeed, Carlier et al. (20), Gnesin et al. (19), and Surti et al. (21) proposed, respectively, a reduction factor of 2.8 between a Vision 450 and a mCT, 3.12 and 4–6 between a Vision 600 and a mCT, respectively. Nevertheless, the extrapolation of phantom data remains anyways difficult in human clinical daily practice and only few studies have evaluated the clinical impact of digital PET/CT system in patients. Lopez-Mora et al. (22) compared image quality and lesion detection capability between a digital and an analog PET/CT system. One hundred patients sequentially underwent both a digital and an analog PET/CT with a mean delay of 50 +/– 14 min between the two image acquisitions. The digital system allowed a significantly better image quality and a greater lesion detectability than the analog system. In the same cohort, Fuentes-Ocampo et al. (23) assessed whether digital PET/CT impacts the quantification of SUVmax in target lesions and in reference regions (liver and mediastinal blood pool) in comparison to analog PET/CT. They concluded that SUVmax of target lesions and mediastinal blood pool measured by the digital system were significantly higher than those obtained by the analog one whereas liver mean SUVmax did not differ between the two PET/CT systems. More recently, van Sluis et al. (24) evaluated the image quality and semiquantitative analysis between a digital PET/CT system (Biograph Vision) and its analog predecessor (Biograph mCT). The authors concluded that the Biograph Vision outperforms the Biograph mCT in terms of image quality, lesion demarcation, and signal-to-noise ratio without any significant difference in semi-quantitatively assessment in a population of 20 patients. Lately, Kim et al. (25) confirmed also in a small cohort (30 patients) that digital PET/CT (Discovery MI; GE Healthcare) provides improved image quality and lesion detectability compared to a standard PET/CT (Biograph mCT, Siemens Healthineers). The mean time delay between both scans was shorter than that in Van Sluis study (17 vs. 37 min) (24, 25). The authors stressed as limitation of their studies the impossibility of performing both acquisitions at the same time and especially the impossibility of taking into account the evolution of FDG biodistribution between the two acquisitions (23, 24). In order to overcome this limitation, we analyzed 4 PET datasets acquired at the same time. We also evaluated the improvement of image quality according to BMI. Indeed in a recent study published by Hatami et al. (26), the authors concluded that the most impactful difference in scan time, dosimetry and FDG dose was observed in overweight patients. Moreover, we found in our cohort that, whatever the BMI, digital PET/CT system improved the image quality.

Thanks to an improved image quality and a better detectability, the use of digital PET/CT is expected to improve patient management enabling better characterization and detection of lesions previously unseen when using analog systems (27). The improvement of the confidence index described in our study could particularly lead to a decrease in the proportion of uptakes deemed equivocal. The increase of detected lesions might lead as well to improve staging accuracy and prognostic value, by example an earlier diagnosis of small metastatic lesions.

In the other hand, while image quality and detectability is improved by digital PET systems with an equal or reduced acquisition duration, this improvement could probably be used as well to optimize administered activity. Indeed in clinical practice, taking into account particular circumstances (screening, pediatric population, painful patient, claustrophobia, etc.), these improvements have to be considered to determine the most optimized image quality with the lowest radiation and shortest acquisition duration.

Our study presents some limitations. Firstly, we compared three digital datasets to an “analog-like” dataset, which was actually a simulated analog dataset. This “analog-like” dataset may differ slightly from an actual analog system. However, we performed a phantom study in order to minimize the differences between the analog and the analog-like images and validate the analog-like reference. Secondly, physicians who reviewed all PET reconstructions read all 4 datasets simultaneously and knew which parameters were used for each dataset. This could have impacted the assessment of quality indices, by directly comparing all datasets together. Finally, the clinical impact of new uptakes or lesions that were detected only on highest quality images cannot be determined in this study.

## Conclusion

In summary, our study demonstrated in a large cohort an improvement of image quality and lesion detectability with a digital PET/CT system. Future studies should assess the impact of these changes on clinical management.

## Data Availability Statement

The raw data supporting the conclusions of this article will be made available by the authors, without undue reservation.

## Ethics Statement

The studies involving human participants were reviewed and approved by the ethics committee of Brest University Hospital reviewed and approved the protocol of our retrospective study (approval number B2020CE.22). All patients provided written informed consent. The patients/participants provided their written informed consent to participate in this study.

## Author Contributions

OD, NK, and SQ interpetated the different PET/CT. DB performed all the reconstructions. NK prepared tables and figures. OD performed the statistical analysis. SQ wrote the manuscript. PYS and SQ designed the study. RA, PYLR, and PR corrected the manuscript. All authors contributed to the article and approved the submitted version.

## Conflict of Interest

The authors declare that the research was conducted in the absence of any commercial or financial relationships that could be construed as a potential conflict of interest.
